# Supplementary material for: Profiling the inhibitory receptors LAG-3, TIM-3, and TIGIT in renal cell carcinoma reveals malignancy
Source: Nat Commun. 2021 Sep 20;12:5547. doi: 10.1038/s41467-021-25865-0 (PMC8452744; doi:10.1038/s41467-021-25865-0)
Supplement: Supplementary file 1 — Supplementary Information [file 41467_2021_25865_MOESM1_ESM.pdf]

Supplementary Information (on-line only)

Table of contents

Supplementary Figure 1. 2
Supplementary Figure 2 3
Supplementary Figure 3 4
Supplementary Figure 4 5
Supplementary Figure 5 5

Supplementary Table 1 6
Supplementary Table 2 6
Supplementary Table 3 7
Supplementary Table 4 7
Supplementary Table 5 8

Supplementary Fig 1

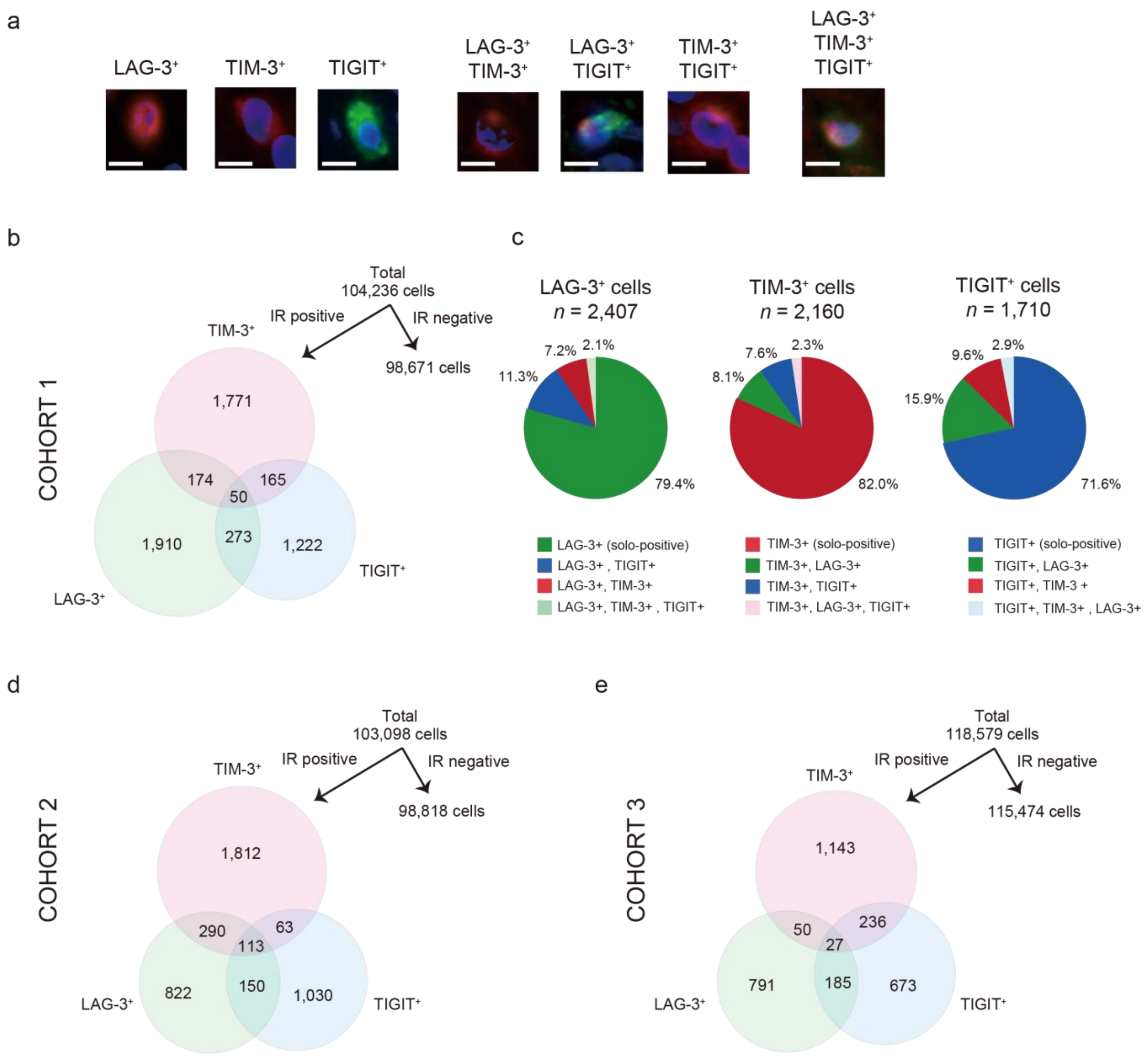

Supplementary Fig 1. Cellular relationship of LAG-3, TIM-3, and TIGIT expression in human RCC tumours by multiplexed immunofluorescence staining. a Cell-by-cell images of each defined cell phenotype displaying multi-colour immunolabelling for LAG-3, TIM-3, and TIGIT together with DAPI. Scale bars, 10 µm (white) **b** Venn diagram depicting the cellular overlap of the three IR expressions with multi-colour staining for LAG-3, TIM-3, and TIGIT in COHORT 1 samples. **c** Percent distributions of different IR expressions in inferred IR-positive cells in **b**. **d-e** Venn diagram depicting the cellular overlap of the three IR expressions with multi-colour staining for LAG-3, TIM-3, and TIGIT in COHORT 2 (**d**) and COHORT 3 (**e**) samples.

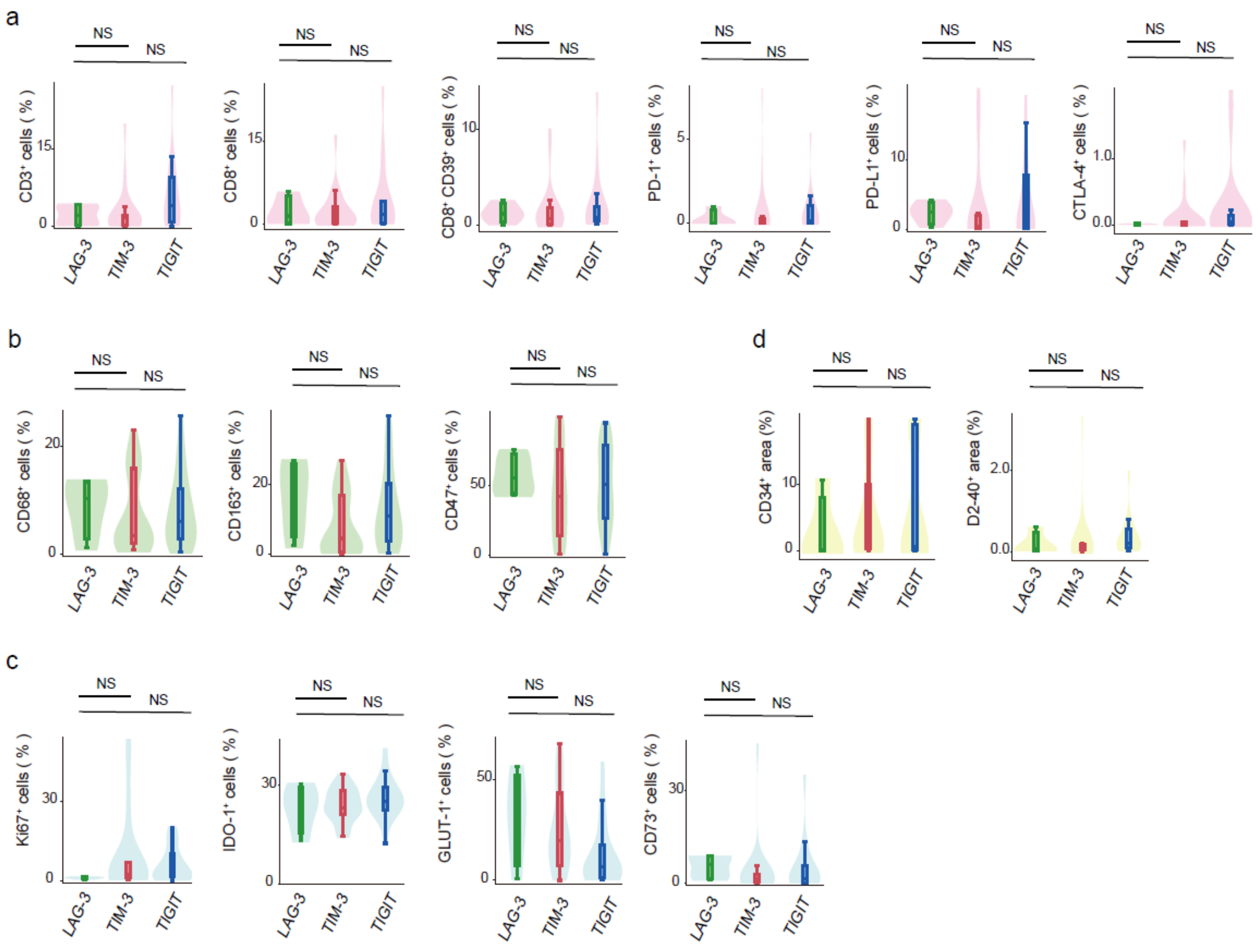

Supplementary Fig 2. Phenotypic signatures of the new IRs (LAG-3, TIM-3, and TIGIT) and the tumour immune microenvironment in human non-ccRCC tumours. a-d Violin and box plots of acquired immunity (**a**), innate immunity (**b**), inhibitory tumour metabolism (**c**), and vascular attribute (**d**) signatures from 41 human non-ccRCC samples. The lines within the boxes represent the medians, the upper and lower ends of the boxes represent the upper and lower quartiles, and the bars represent the minimum and maximum values in 1.5 times the IQR. *p* values were determined with a two-tailed Mann–Whitney U-test. Source data are provided as a Source data file.

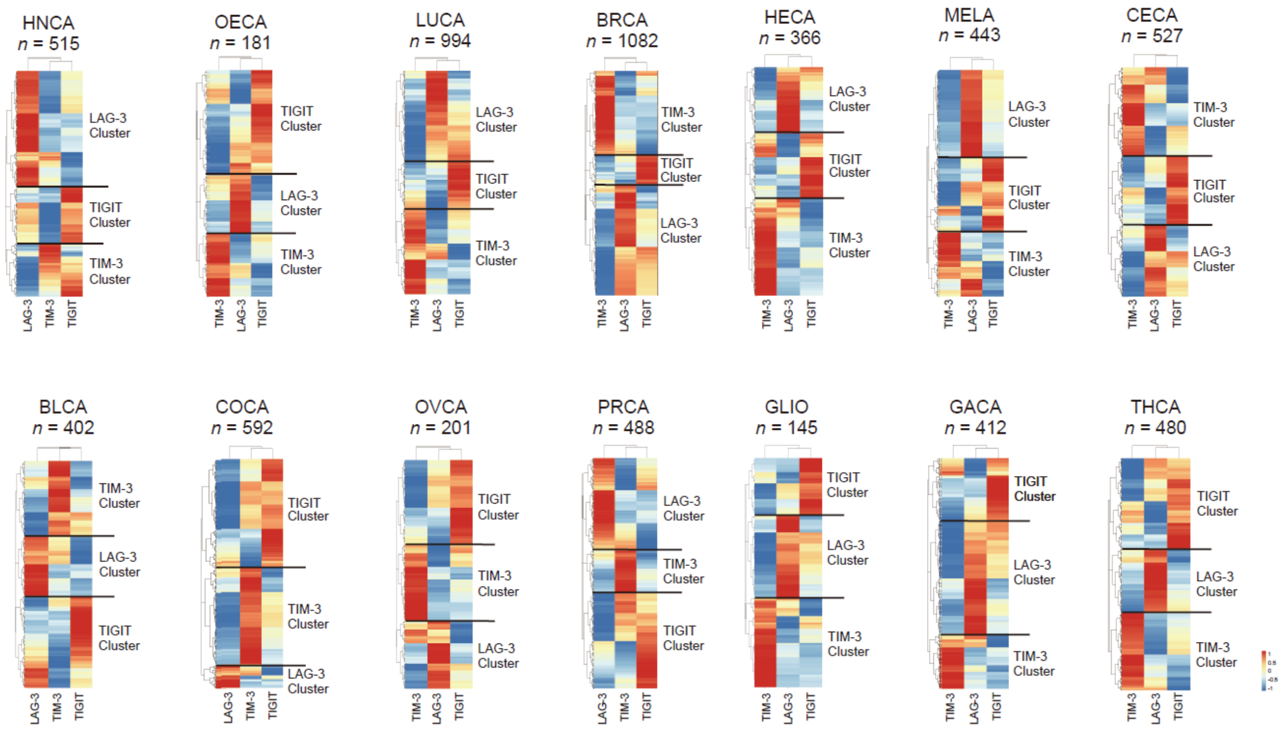

Supplementary Fig 3. A pan-cancer analysis of LAG-3, TIM-3, and TIGIT gene expression levels. Hierarchical clustering heatmap (low, blue; high, red) using inferred IR signatures from RNA-seq data analysis of 14 different cancer types in the TCGA dataset.[41)]

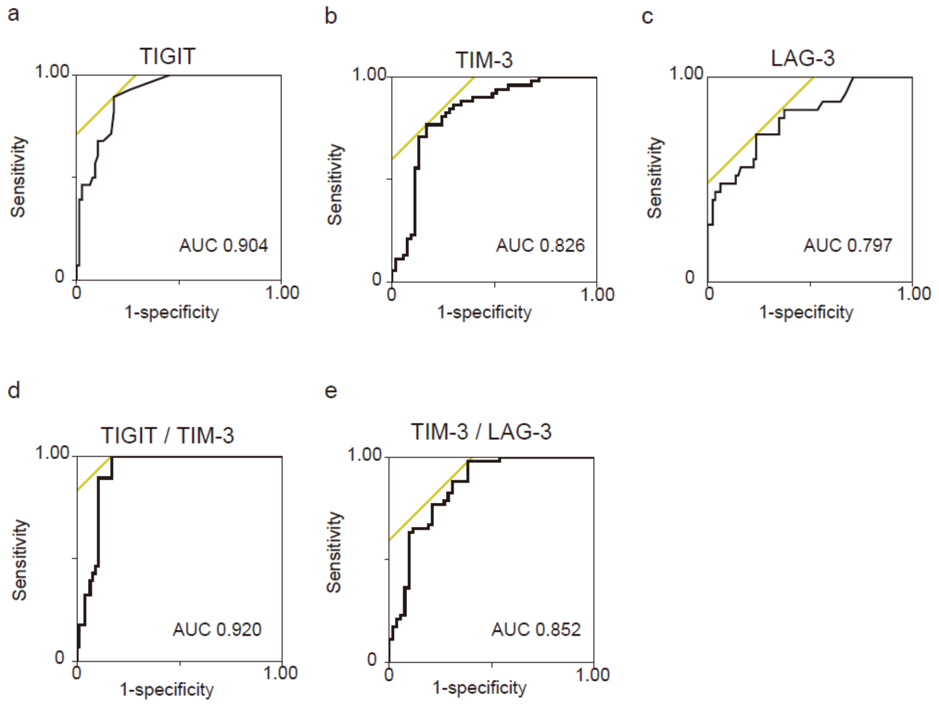

Supplementary Fig 4. ROC-AUC analysis discriminating the phenotypic signatures of the new IRs (LAG-3, TIM-3, and TIGIT) by immunohistochemistry in COHORT 1. a-e Receiver operating characteristic (ROC) curves for TIGIT (a), TIM-3 (b) and LAG-3 (c) expression, the ratio of TIGIT/TIM-3 (d) and TIM-3/LAG-3 (e) by immunohistochemistry for the new IRs clusters.

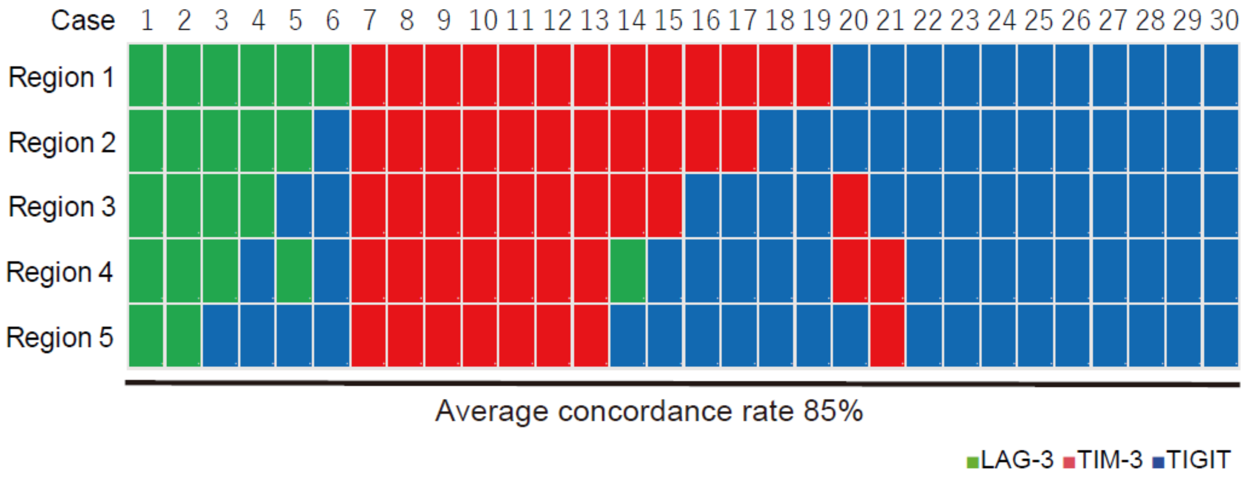

Supplementary Fig 5. Consistency in IR profiles within the same tumour obtained by multi-region samplings from a single tumour in 30 primary ccRCC patients in COHORT 1.

Supplementary Table 1. Parameters associated with postoperative disease recurrence and overall mortality in primary ccRCC after adjusting for confounders with univariable and multivariable Cox regression analyses in COHORT 1.

Variable	Recurrence-free survival						Overall survival					
	Univariate	Multivariate					Univariate	Multivariate				
	p value	HR	95% CI			*p* value	*p* value	HR	95% CI			*p* value
Age	0.015	1.03	1.00	-	1.05	0.043	0.001	1.05	1.02	-	1.09	0.005
Gender (male vs. female)	0.615					0.691	0.222					0.642
Nuclear grade (G3/4 vs. G1/2)	< 0.001	2.32	1.26	-	4.30	0.007	< 0.001	3.39	1.49	-	7.72	0.004
Pathological T stage (pT3/4 vs. pT1/2)	< 0.001	1.97	1.11	-	3.48	0.020	< 0.001					0.341
Venous invasion (yes vs. no)	0.087					0.779	< 0.001					0.200
Tumour size	< 0.001	1.01	1.00	-	1.02	0.022	< 0.001	1.01	1.00	-	1.02	0.047
IR profile	0.291					0.656	0.028					0.037
LAG-3 vs. TIM-3	0.930					0.543	0.047	2.15	1.03	-	4.46	0.041
LAG-3 vs. TIGIT	0.146					0.391	0.040	4.74	0.63	-	35.71	0.131

Supplementary Table 2. Clinicopathological characteristics associated with the phenotypic signatures of the new IRs (LAG-3, TIM-3, and TIGIT) in 47 metastatic ccRCC tumour samples (COHORT 2). ***p*** **value was determined with a two-tailed Fisher's exact test.**

Characteristic	All patients (*n* = 47)	IR profile			*p* value
		LAG-3 *n* = 14 (30%)	TIM-3 *n* = 22 (47%)	TIGIT *n* = 11 (23%)	
Localisation of distant metastasis, no. (%):					0.071
Bone	18 (38%)	9 (64%)	5 (23%)	4 (36%)	
Viscera	11 (23%)	1 (7%)	8 (36%)	2 (18%)	
Lung	8 (17%)	2 (14%)	2 (9%)	4 (36%)	
Brain	4 (9%)	0 (0%)	4 (18%)	0 (0%)	
Others	6 (13%)	2 (14%)	3 (14%)	1 (9%)	

Supplementary Table 3. Clinicopathological characteristics associated with the phenotypic signatures of the new IRs (LAG-3, TIM-3, and TIGIT) in 41 primary non-ccRCC tumour samples (COHORT 3). ***p*** **value was determined with a two-tailed Fisher's exact test.**

Characteristic	All patients (*n* = 41)	IR profile: LAG-3 *n* = 4 (10%)	IR profile: TIM-3 *n* = 20 (49%)	IR profile: TIGIT *n* = 17 (41%)	*p* value
Histological type, no (%):					0.213
Papillary	12 (29%)	2 (50%)	4 (20%)	6 (35%)	
Chromophobe	12 (29%)	0 (0%)	10 (50%)	2 (12%)	
Sarcomatoid	8 (20%)	1 (25%)	3 (15%)	4 (24%)	
Xp11 translocation	7 (17%)	1 (25%)	2 (10%)	4 (24%)	
Collecting duct	2 (5%)	0 (0%)	1 (5%)	1 (6%)	

Supplementary Table 4. Parameters associated with postoperative disease recurrence and overall mortality in primary ccRCC after adjusting for confounders with univariable and multivariable Cox regression analyses in COHORT 4.

Variable	Recurrence-free survival				Overall survival			
	Univariate	Multivariate			Univariate	Multivariate		
	p value	HR	95% CI	*p* value	*p* value	HR	95% CI	*p* value
Age	0.862			0.690	0.132			0.508
Gender (male vs. female)	0.960			0.852	0.518			0.671
Nuclear grade (G3/4 vs. G1/2)	< 0.001			0.072	<0.001			0.688
Pathological T stage (pT3/4 vs. pT1/2)	< 0.001	5.13	2.23 - 11.76	<0.001	0.001	2.61	0.87 - 7.81	0.086
Venous invasion (yes vs. no)	< 0.001			0.243	<0.001	4.90	1.60 - 14.93	0.005
Tumour size	< 0.001	1.03	1.01 - 1.05	0.002	0.001			0.109
IR profile	0.052			0.167	0.003			0.009
LAG-3 vs. TIM-3	0.021			0.086	0.004	4.81	1.60 - 15.87	0.010
LAG-3 vs. TIGIT	0.097			0.747	0.013	4.69	1.28 - 17.24	0.020

Supplementary Table 5. List of antibodies used for immunohistochemical studies.

Antibodies	Source	Cat #	Dilution
Rabbit monoclonal anti-LAG-3	Abcam	ab209236	1/50-1/500
Rabbit polyclonal anti-TIM-3	Abcam	ab185703	1/100
Mouse monoclonal anti-TIGIT	DIANOVA	#DIA-TG1–M	1/100
Rabbit monoclonal anti-CD3	NICHIREI	#413591	Diluted
Mouse monoclonal anti-CD8	NICHIREI	#413211	1/50
Rabbit monoclonal anti-CD39	Abcam	ab223842	1/1000
Mouse monoclonal anti-PD-1	Cell Signaling Technology	#43248	1/200
Rabbit monoclonal anti-PD-L1	Cell Signaling Technology	#13684	1/200
Mouse monoclonal anti-CTLA-4	Abcam	ab227709	1/100
Mouse monoclonal anti-CD68	NICHIREI	413791	Diluted
Rabbit monoclonal anti-CD163	Abcam	ab182422	1/500
Rabbit polyclonal anti-CD47	Abcam	ab175388	1/500
Ki67	DAKO	M7240	1/50
IDO-1	Origene	UM 570091	1/100
Rabbit monoclonal anti-GLUT-1	Abcam	ab115730	1/500
Rabbit monoclonal anti-CD73	Abcam	ab133582	1/100
Mouse monoclonal anti-CD34	NICHIREI	#413361	1/100
Mouse monoclonal anti-D2-40	NICHIREI	#413451	Diluted
