# Supplementary material for: Profiling the inhibitory receptors LAG-3, TIM-3, and TIGIT in renal cell carcinoma reveals malignancy
Source: Nat Commun. 2021 Sep 20;12:5547. doi: 10.1038/s41467-021-25865-0 (PMC8452744; doi:10.1038/s41467-021-25865-0)
Supplement: Supplementary file 3 — Description of Additional Supplementary Files [file 41467_2021_25865_MOESM3_ESM.pdf]

Description of Additional Supplementary Files

File Name: Supplementary Data 1
Description: Clinical and pathological data of COHORT1

File Name: Supplementary Data 2
Description: Clinical and pathological data of COHORT4
