# Supplementary material for: Profiling the inhibitory receptors LAG-3, TIM-3, and TIGIT in renal cell carcinoma reveals malignancy
Source: Nat Commun. 2021 Sep 20;12:5547. doi: 10.1038/s41467-021-25865-0 (PMC8452744; doi:10.1038/s41467-021-25865-0)
Supplement: Supplementary file 6 — Reporting Summary [file 41467_2021_25865_MOESM6_ESM.pdf]

nature research

Corresponding author(s): Nobuyuki Tanaka

Last updated by author(s): Aug 9, 2021

Reporting Summary

Nature Research wishes to improve the reproducibility of the work that we publish. This form provides structure for consistency and transparency in reporting. For further information on Nature Research policies, see our Editorial Policies and the Editorial Policy Checklist.

Statistics

For all statistical analyses, confirm that the following items are present in the figure legend, table legend, main text, or Methods section.

n/a	Confirmed	
☐	☒	The exact sample size (*n*) for each experimental group/condition, given as a discrete number and unit of measurement
☐	☒	A statement on whether measurements were taken from distinct samples or whether the same sample was measured repeatedly
☐	☒	The statistical test(s) used AND whether they are one- or two-sided<br>*Only common tests should be described solely by name; describe more complex techniques in the Methods section.*
☐	☒	A description of all covariates tested
☐	☒	A description of any assumptions or corrections, such as tests of normality and adjustment for multiple comparisons
☐	☒	A full description of the statistical parameters including central tendency (e.g. means) or other basic estimates (e.g. regression coefficient) AND variation (e.g. standard deviation) or associated estimates of uncertainty (e.g. confidence intervals)
☐	☒	For null hypothesis testing, the test statistic (e.g. *F*, *t*, *r*) with confidence intervals, effect sizes, degrees of freedom and *P* value noted<br>*Give P values as exact values whenever suitable.*
☒	☐	For Bayesian analysis, information on the choice of priors and Markov chain Monte Carlo settings
☐	☒	For hierarchical and complex designs, identification of the appropriate level for tests and full reporting of outcomes
☒	☐	Estimates of effect sizes (e.g. Cohen's *d*, Pearson's *r*), indicating how they were calculated

Our web collection on statistics for biologists contains articles on many of the points above.

Software and code

Policy information about availability of computer code

Data collection	No software was used for data collection
Data analysis	HistoQuest software, TissueGnostics, Vienna, Austria, inForm software ver. 2.4 (PerkinElmer), GenomeJack (Mitsubishi Space Software, Tokyo, Japan), R statistical language version 3.6.1 (R Foundation for Statistical Computing, Vienna, Austria), SPSS version 24.0 (IBM-SPSS Inc., Tokyo, Japan) and JMP version 15.0 (SAS Institute Inc., Cary, NC, USA).

For manuscripts utilizing custom algorithms or software that are central to the research but not yet described in published literature, software must be made available to editors and reviewers. We strongly encourage code deposition in a community repository (e.g. GitHub). See the Nature Research guidelines for submitting code & software for further information.

Data

Policy information about availability of data

All manuscripts must include a data availability statement. This statement should provide the following information, where applicable:

- Accession codes, unique identifiers, or web links for publicly available datasets
- A list of figures that have associated raw data
- A description of any restrictions on data availability

The DNA-seq data have been deposited in the SRA database under accession code PRJNA739032. (http://www.ncbi.nlm.nih.gov/bioproject/739032.) The TCGA dataset for kidney renal clear cell carcinoma in Fig 5 was downloaded from the cBioPortal (https://www.cbioportal.org/study/summary?id=kirc_tcga_pan_can_atlas_2018; access date: April, 2020) (Reference, 27). The SATO dataset in Fig 5 was downloaded from the EGA database (https://ega-archive.org/datasets/EGAD00001000597; access date: April, 2020) (Reference, 29). Remaining TCGA datasets from 14 cancer types in Supplementary Fig 3 were downloaded from the cBioPortal (https://www.cbioportal.org/study/summary?id=hnsc_tcga_pan_can_atlas_2018; https://www.cbioportal.org/study/summary?id=esca_tcga_pan_can_atlas_2018; https://www.cbioportal.org/study/summary?id=lusc_tcga_pan_can_atlas_2018%2Cluad_tcga_pan_can_atlas_2018; https://

www.cbioportal.org/study/summary?id=brca_tcga_pan_can_atlas_2018; https://www.cbioportal.org/study/summary?id=lihc_tcga_pan_can_atlas_2018; https://www.cbioportal.org/study/summary?id=skcm_tcga_pan_can_atlas_2018; https://www.cbioportal.org/study/summary?id=ucec_tcga_pan_can_atlas_2018; https://www.cbioportal.org/study/summary?id=blca_tcga_pan_can_atlas_2018; https://www.cbioportal.org/study/summary?id=coadread_tcga_pan_can_atlas_2018; https://www.cbioportal.org/study/summary?id=ov_tcga_pan_can_atlas_2018; https://www.cbioportal.org/study/summary?id=prad_tcga_pan_can_atlas_2018; https://www.cbioportal.org/study/summary?id=gbm_tcga_pan_can_atlas_2018; https://www.cbioportal.org/study/summary?id=stad_tcga_pan_can_atlas_2018; https://www.cbioportal.org/study/summary?id=thca_tcga_pan_can_atlas_2018; access date April, 2021) (Reference, 41). The source data underlying all Figures and Supplementary Figures are provided as a Source Data file. All the other data supporting the findings of this study are available within the article and its Supplementary Information files. Due to privacy concerns restricted by the ethics, clinical data of individual patients (i.e., indirect identifiers patient age and gender in Supplementary Data 1-2) will be shared, after proper de-identification, upon reasonable request to the corresponding author from colleagues who want to analyse in deep our findings. Also, remaining data will be shared upon reasonable request to the corresponding author.

Field-specific reporting

Please select the one below that is the best fit for your research. If you are not sure, read the appropriate sections before making your selection.

- [x] Life sciences
- [ ] Behavioural & social sciences
- [ ] Ecological, evolutionary & environmental sciences

For a reference copy of the document with all sections, see nature.com/documents/nr-reporting-summary-flat.pdf

Life sciences study design

All studies must disclose on these points even when the disclosure is negative.

Sample size	Sample size was determined by the practical limitations of the protocol utilized. No statistical estimation of sample size was performed.
Data exclusions	Data were not excluded from analyses.
Replication	The experiment was performed one time because of using human sample. Analyses were reliably reproduced.
Randomization	Not performed because this study was retrospective.
Blinding	Analyses were performed independently of patient characteristics and clinical data.

Reporting for specific materials, systems and methods

We require information from authors about some types of materials, experimental systems and methods used in many studies. Here, indicate whether each material, system or method listed is relevant to your study. If you are not sure if a list item applies to your research, read the appropriate section before selecting a response.

Materials & experimental systems

n/a	Involved in the study	
[ ]	[x]	Antibodies
[x]	[ ]	Eukaryotic cell lines
[x]	[ ]	Palaeontology and archaeology
[x]	[ ]	Animals and other organisms
[ ]	[x]	Human research participants
[x]	[ ]	Clinical data
[x]	[ ]	Dual use research of concern

Methods

n/a	Involved in the study	
[x]	[ ]	ChIP-seq
[x]	[ ]	Flow cytometry
[x]	[ ]	MRI-based neuroimaging

Antibodies

Antibodies used	All information regarding antibodies is included in the "Immunohistochemistry" section and "Supplementary Table 5".<br>Clone name of each monoclonal antibody used in the study.<br>Rabbit monoclonal anti-LAG-3, EPR20261, Mouse monoclonal anti-TIGIT, TG1,Rabbit monoclonal anti-CD3, SP7, Mouse monoclonal anti-CD8, C8/144B, Rabbit monoclonal anti-CD39, EPR20627, Mouse monoclonal anti-PD-1, EH33, Rabbit monoclonal anti-PD-L1, E1L3N, Mouse monoclonal anti-CTLA-4, SP355, Mouse monoclonal anti-CD68, PG-M1, Rabbit monoclonal anti-CD163, EPR19518, Mouse monoclonal anti-Ki67, MIB-1, Mouse monoclonal anti-IDO-1, UMAB126, Rabbit monoclonal anti-GLUT-1, EPR3915, Rabbit monoclonal anti-CD73, EPR6114, Mouse monoclonal anti-CD34, NU-4A1, Mouse monoclonal anti-D2-40, D2-40
Validation	Validation of the antibodies was based on the manufacturer's information.<br>Rabbit monoclonal anti-LAG-3 Human tonsil and Hodgkin's lymphoma tissues.<br>Rabbit polyclonal anti-TIM-3 Human lung cancer tissue, human tonsil tissue and mouse spleen tissue.<br>Mouse monoclonal anti-TIGIT Human tonsil<br>Rabbit monoclonal anti-CD3 Human tonsil

Mouse monoclonal anti-CD8 Human lymph node
Rabbit monoclonal anti-CD39 Human colon carcinoma, spleen and liver tissues; Mouse spleen tissue.
Mouse monoclonal anti-PD-1 Human colon adenocarcinoma
Rabbit monoclonal anti-PD-L1 Human non-small cell lung carcinoma
Mouse monoclonal anti-CTLA-4 Human tonsil tissue
Mouse monoclonal anti-CD68 Human tonsil
Rabbit monoclonal anti-CD163 Human liver, tonsil and placenta tissue.; human breast carcinoma tissue; Mouse liver and spleen tissue. Rat liver, achilles and muscle tissues
Rabbit polyclonal anti-CD47 human placenta and human lung cancer tissues
Mouse monoclonal anti-Ki67 Human tonsil
Mouse monoclonal abti-IDO-1 Human spleen
Rabbit monoclonal anti-GLUT-1 Rat kidney tissue; mouse liver tissue; human lung carcinoma, cervical carcinoma, colon carcinoma, liver, colon, kidney carcinoma
Rabbit monoclonal anti-CD73 Human lung carcinoma, Human tonsil tissue ICC/IF: A375 cells
Mouse monoclonal anti-CD34 Human placenta
Mouse monoclonal anti-D2-40 Human duodenum

Human research participants

Policy information about studies involving human research participants

Population characteristics	This study involved 289 participants. The details of participants are as follows: COHORT 1, primary ccRCC tumours treated surgically (n = 105, Table 1); COHORT 2, ccRCC tumour metastases diagnosed histologically (n = 47: lung, 8; bone, 18; viscera, 11, brain, 4, and others, 6; Supplementary Table 2); COHORT 3, primary non-ccRCC tumours (n = 41: papillary, 12; chromophobe, 12; sarcomatoid, 8; Xp11.2 translocation, 7; and collecting duct, 2; Supplementary Table 3) and COHORT 4, primary ccRCC tumours treated surgically (n = 96, Table 2).
Recruitment	After approval from the Institutional Review Board, tumour samples obtained from 1999-2017 were randomly collected. These samples were residual from a clinical examination without using any identifiable information of the individuals or the application of any intervention. Both written informed consent or passive (opt-out) informed consent procedures have been applied to the experimental use of human samples. Participation in the study was optional. Opt-out informed consent from patients was obtained by exhibiting the research information on our department website (Department of Urology, Keio University Hospital, Tokyo, Japan). All participant patients or families of deceased patients could withdraw consent by contacting the researcher with a 24-hour phone number. The need to obtain written informed consent was waived if patients had finished their follow-up or had died, due to the study's observational nature and the urgent need for cancer patient care. This was approved and reviewed by the Research Ethics Committee of Keio University, in accordance with the ethical guidelines for Medical and Health Research Involving Human Subjects (Public Notice of the Ministry of Education, Culture, Sports, Science and Technology and the Ministry of Health, Labor and Welfare as of July 2018; https://www.lifescience.mext.go.jp/files/pdf/n2181_01.pdf).
Ethics oversight	The Research Ethics Committee of Keio University (Approval No-20180098 and 20190059)

Note that full information on the approval of the study protocol must also be provided in the manuscript.
